# Integrated Method for Personal Thermal Comfort Assessment and Optimization through Users’ Feedback, IoT and Machine Learning: A Case Study †

**DOI:** 10.3390/s18051602

**Published:** 2018-05-17

**Authors:** Francesco Salamone, Lorenzo Belussi, Cristian Currò, Ludovico Danza, Matteo Ghellere, Giulia Guazzi, Bruno Lenzi, Valentino Megale, Italo Meroni

**Affiliations:** 1 ITC-CNR, Construction Technologies Institute-National Research Council of Italy, Lombardia St., 49-20098 San Giuliano M.se, Italy; belussi@itc.cnr.it (L.B.); danza@itc.cnr.it (L.D.); ghellere@itc.cnr.it (M.G.); guazzi@itc.cnr.it (G.G.); meroni@itc.cnr.it (I.M.); 2 SCS, SoftCare Studios Srls, Franco Sacchetti St., 52-00137 Roma, Italy; c.curro@tommigame.com (C.C.); b.lenzi@tommigame.com (B.L.); info@softcarestudios.com (V.M.)

**Keywords:** indoor thermal comfort, wearable, nearable, IoT, machine learning, parametric models

## Abstract

Thermal comfort has become a topic issue in building performance assessment as well as energy efficiency. Three methods are mainly recognized for its assessment. Two of them based on standardized methodologies, face the problem by considering the indoor environment in steady-state conditions (PMV and PPD) and users as active subjects whose thermal perception is influenced by outdoor climatic conditions (adaptive approach). The latter method is the starting point to investigate thermal comfort from an overall perspective by considering endogenous variables besides the traditional physical and environmental ones. Following this perspective, the paper describes the results of an in-field investigation of thermal conditions through the use of nearable and wearable solutions, parametric models and machine learning techniques. The aim of the research is the exploration of the reliability of IoT-based solutions combined with advanced algorithms, in order to create a replicable framework for the assessment and improvement of user thermal satisfaction. For this purpose, an experimental test in real offices was carried out involving eight workers. Parametric models are applied for the assessment of thermal comfort; IoT solutions are used to monitor the environmental variables and the users’ parameters; the machine learning CART method allows to predict the users’ profile and the thermal comfort perception respect to the indoor environment.

## 1. Introduction

Thermal Comfort (TC) is defined as the psychophysical satisfaction of an individual immersed in a thermal environment [1]. As described in the EN ISO 7730:2005 standard [2], TC is influenced by six factors [3], summarized in two categories: four objective variables: air temperature (T_air_), relative humidity (RH), air velocity (V_air_), and mean radiant temperature (T_rad_) and two subjective variables: metabolic activity and clothing. Over the years, several studies have highlighted how the thermal sensation of users further depends on factors linked to human characteristics, e.g., age, gender, pathologies, etc. [4,5,6,7,8,9,10,11]. Adaptive approaches, on the basis of the subjective response of individuals to thermal stimuli, have been proposed in order to include these factors in thermal assessment process. At present, there are two main approaches to assess the TC: the thermal balance or rational method and the adaptive method (AM) [12,13]. The former is mainly based on the tests carried out by Fanger on a sample of 1296 Danish students in specific climate controlled chambers under steady state conditions [14,15]. The participants were dressed in standard clothing and performed standard activities. The only variable was the exposure to different environmental conditions. Starting from these experiments Fanger defined an equation that describes the TC such as the balance between the current thermal flow on human body, in a given environment, and that corresponding to an optimal comfort in relation to a specific activity. This equation allows to define the so called Predicted Mean Vote (PMV) index. Therefore, the Predicted Percentage of Dissatisfied (PPD) index expresses the percentage of dissatisfied as a function of thermal sensation perceived by the users and was calculated as a function of the PMV. Both the indices are used to define the TC level of an indoor environment. The indices are commonly adopted for the assessment of indoor spaces equipped with HVAC systems or in naturally ventilated buildings, if the expectancy factor is known [15,16]. Furthermore the EN 15251:2007 considers the PMV level to define the operative temperature (T_o_) ranges for energy calculations [17].

In the adaptive approaches, the main object of the analysis is the final users’ satisfaction in order to optimize the thermal acceptability of the indoor environment. The AM described by the ASHRAE 55:2017 standard [18] couples the T_o_ with the mean outdoor temperature. More generally, the user’s adaptation to thermal stimuli can be divided into three categories: behavioral, physiological and psychological [19,20,21,22,23]. The former includes all conscious and unconscious modifications that an individual carries out by changing the heat and mass flows to regulate the thermal balance of the own body (change of clothing, use of air-conditioning systems, etc.). The physiological adaptation includes the changes in the physiological response with respect to the environmental exposure (acclimatization). The latter represents a wrong perception or a response to stimuli linked to past experiences.

Several studies [24,25,26,27] investigated TC in a multi-domain approach modifying the comfort models according to subjective assessment. In particular, in [23] a pulse sensor is used in Ambient Assisted Living to identify the heart rate [bpm] (HR) and consequently the metabolic rate following the algorithm presented in ISO 8996 [28] as a key parameter in the determination of the PMV. In [25] the quantitative estimation of the thermal comfort level in sports facilities is described with the aim of supporting the development of comfort-based metering and energy control systems. In [26] the authors propose a data-driven approach for real time prediction of individual thermal comfort level based on the classical objective and subjective data without the use of biometric values. In [27] the authors define a method to predict individuals’ thermal preference using occupant heating and cooling behavior and machine learning (ML) techniques.

The state-of-art demonstrates how the assessment of TC in the built environment is still a challenge. The design of comfortable and healthy indoor environments should be the goal of professionals. Researchers constantly investigate this issue in order to define new methodologies and tools for the prediction and assessment of TC. The current trend is the deep evaluation of users’ perception. The application of Internet of Things (IoT) solutions and advanced algorithms is becoming an alternative to traditional methods, both for monitoring and calculation process.

Following this path, the paper describes a framework [29] for the assessment of the thermal conditions of users through the analysis of specific psychophysical conditions, the application of IoT-based solutions, the assessment of user’s feedback, the use of parametric models and ML techniques; the methodology overcomes the limitation of physical-based models by considering several factors related to human sensation and the complex state of mind that interacts in TC perception [30]. A merit of the proposed method is the detection of internal environmental variables close to users, in addition to the biometric parameters. In this way the overall personal thermal conditions of each user are investigated by considering the actual sensation and the external influencing forces. The method is the basis for the development of individual control of environmental variables.

The Thermal Sensation Vote (TSV) expressed by the user through a web-based survey are compared with the indices provided by the Standards EN ISO 7730:2005 and ASHRAE 55:2017, PMV and the Graphic Comfort Zone Method (GCZM), respectively. The data collected during an experimental campaign in an office building are threaded through ML techniques in order to identify advanced algorithms for the prediction of the user profile and the related optimal TC conditions.

## 2. Description of the Framework

The framework consists of the following parts:

- a monitoring system composed by:
  ○ a nearable device (a term composed by the words “near” and “wearable”) for the monitoring of the environmental parameters nearby the user;
  ○ a wearable device for the monitoring of subjective variables;
- a web-based survey for the detection of users feedback in terms of TSV;
- a parametric model to assess the real TC conditions.

### 2.1. Monitoring Systems

#### 2.1.1. Nearable System

The nearable system applied in the framework is based on low-cost sensors and open-source hardware able to monitor indoor environmental parameters (air temperature, relative humidity, radiant temperature, air velocity, CO_2_ concentration, illuminance level) useful to assess different aspects of the Indoor Environmental Quality (IEQ) [31,32]. The accuracy of the sensors complies the requirements provided by the ISO 7726:1998 [33]. Table 1 reports the characteristics of the sensors used for TC assessment.

The technical characteristics of the low-cost hot wire anemometer are not stated but have been assessed by calibration as reported in [34]. Tests conducted in the 0 ÷ 6 m/s range through the methodology described in [31] allowed to verify the good behavior of the low-cost sensor through direct comparison with a professional one.

#### 2.1.2. Wearable System

The wearable device is the Empatica E4 wristband (Empatica Inc., Cambridge, MA, USA). It is a class II medical device according to the FDA 21 CFR Part 860.3 regulations and is equipped with the following sensors:

- a photoplethysmography (PPG) sensor for the detection of the heart rate (HR) [35];
- an electrodermal activity (EDA) sensor;
- an infrared thermopile;
- a 3-axis accelerometer.

The Empatica E4 wristband was chosen for its verified accuracy [36] and because it is the most practical solution for the purpose of the research. Table 2 reports the characteristics of the sensors used for the acquisition of biometric data.

### 2.2. Web Based Survey

The web-based survey was defined according to the guidelines provided by the ASHRAE 55:2017 standard. It is realized using a Google Forms model allowing a free access at the users. The data are automatically collected in a Google spreadsheet. The information requested at each user are: position occupied in the indoor environment, activity performed, clothing characteristics and thermal sensation on the 7 point scale on general comfort where −3 is equivalent to “cold”, +3 to “hot” and 0 to “neutral” sensation.

The analysis of the survey allowed the identification of the insulation levels related to the clothing, the metabolic rate [37,38,39,40] and the thermal sensation of the individuals. The thermal resistance of the clothing is determined in compliance with the Annex C of the EN ISO 7730:2005 Standard. Each clothing garment is characterized by a specific insulation value, expressed in clo (clothing unit, 1 clo = 0.155 m^2^K/W); the overall thermal resistance is the algebraic sum of the single value of the thermal resistance [41]. The standard provides an additional thermal resistance for sedentary activities due to the type of chair, corresponding to an incremental value equal to 0.17 clo [42]. The standard corrects the static insulation considering a dynamic effect due to both air and body movements [43,44]. Table 3 reports the considered average clothing insulation values for each user.

The common activity of the users provided by the surveys is “typing”. As reported in the Annex B of the EN ISO 7730:2005 standard, for sedentary activity in office, a metabolic rate of 1.2 met is considered.

### 2.3. Parametric Model

The parametric model is realized using Grasshopper, a graphical algorithm editor tightly integrated with Rhino’s 3-D modeling tools [45] and the following plugin:

- Ladybug tools, to assess the TC [46,47] based on PMV and PPD and GCZM;
- TT Toolbox, to import and to export data from a generic database in .csv or .xls formats [48].

## 3. Application of the Framework

### 3.1. First Application and General Data

**Figure 1:**
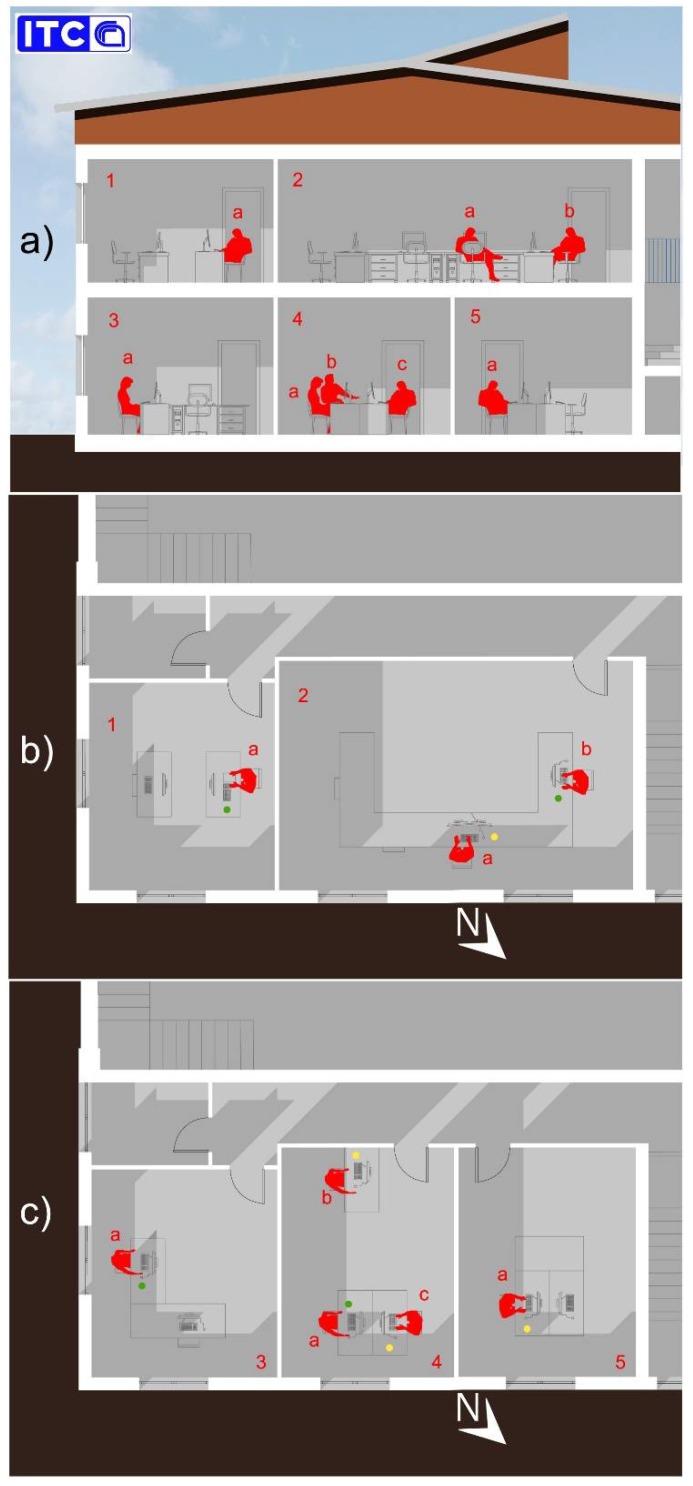
Distribution of the office workstations in the building identified with a number (1–5) and positions of considered users identified with a letter: (**a**) cross section; (**b**) first floor plan; (**c**) ground floor plan. Green and yellow dots point represent the location of the nearable station.

The system was installed on the desktop of eight workstations of a two-story office building located in San Giuliano Milanese, near Milan (Italy) and eight individuals were involved in the survey. The workstations are placed in five offices, three on the ground floor and two on the first floor of the building (Figure 1). Four offices (1, 3, 4, 5) have similar geometrical and morphological characteristics; office n.2 is a double size open space. Offices 1 and 3 have double orientation, East and South-facing; offices 2, 4 and 5 have a single orientation, East-facing. The envelope of the building is characterized by: an external wall consisting of a non-insulated double layer of masonry brick with internal plaster finishing; a concrete basement for floor and a mixed concrete-brick roof; single-pane glass windows with iron frame with the same dimensions. Offices 1 and 3 have two windows, one for each orientation; offices 4 and 5 have a single window each; office 2 has two windows. Each window is equipped with an internal manually-oriented curtain. The heating system consists of radiators placed below the windows. Furthermore, each office is equipped with a manually-controlled reversible heat pump.

**Figure 2:**
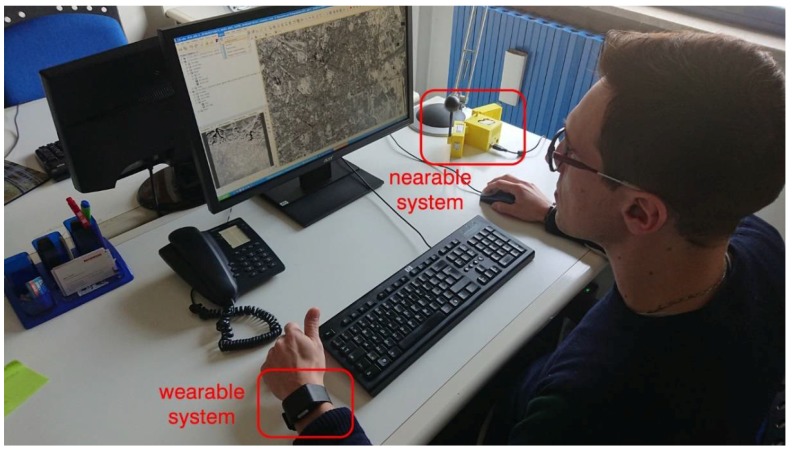
Monitoring system as installed in an office desktop.

The typical installation of the considered monitoring system on the office desktop is shown in Figure 2.

Table 4 reports the areas of the offices, the personal data of the involved users and the periods of the tests. All subjects gave their informed consent for inclusion before they participated in the study.

Table 5 reports the weather data related to the period of the test.

### 3.2. Objective Assessment of Thermal Comfort

The monitored environmental variables have been used along with the users’ subjective variables for the calculation of PMV. The PMV values have been approximated to an integer number (PMV_int_) defined considering the ranges reported in Table 6.

The data collected by the survey are used to verify the difference between the calculated PMV and the TSV. The TSVs are compared with the equivalent PMV_int_ defined considering the average of the environmental value recorded in the previous 5 min. Table 7 shows the percentage difference between the sensation vote expressed by the feedbacks of each user and the related calculated PMV_int_.

The greatest difference between the indices is recorded for users 2a and 2b with a PMV_int_ value higher than 60% of the TSV and for users 4a and 4c with a value greater than 40%.

**Figure 3:**
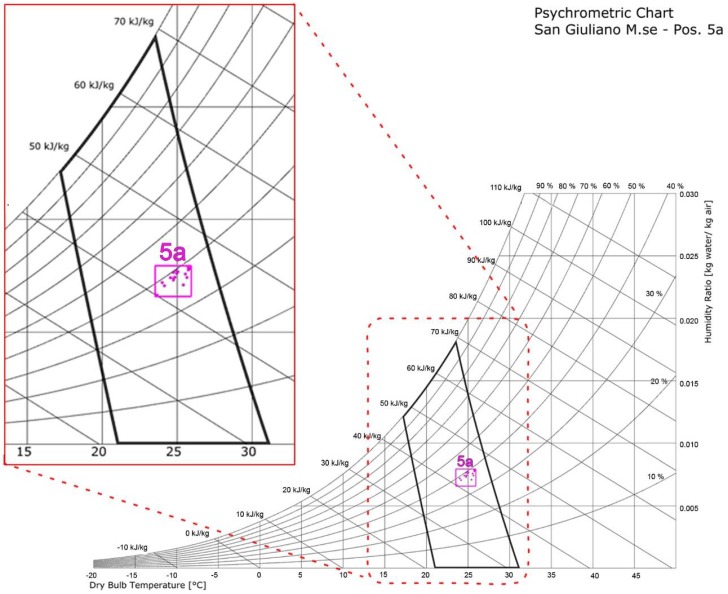
GCZM (black line) and GCZa (pink line) for user 5a.

Moreover, the analysis makes use of the psychrometric chart to identify the comfort zone according to the GCZM and the adapted Graphic Comfort Zone (GCZa), defined as the environmental variables corresponding to TSV equal to 0. Figure 3 shows the comfort zone [49,50] according to the GCZM (black line) and the GCZa (pink line) for users 5a. The black line in Figure 3 is defined as a function of the thermal insulation, the metabolic rate, the radiant temperature and the air velocity. The pink line is based on the environmental data averaged over a period of a minute at the time where the user 5a has provided a TSV equal to 0. The differences in term of comfort zone are quite evident, with a more restricted area in the approach based on the user’s feedback. The new individual comfort zone could be considered in a hypothetical adaptive control and optimization strategy of the office thermal plant system.

**Figure 4:**
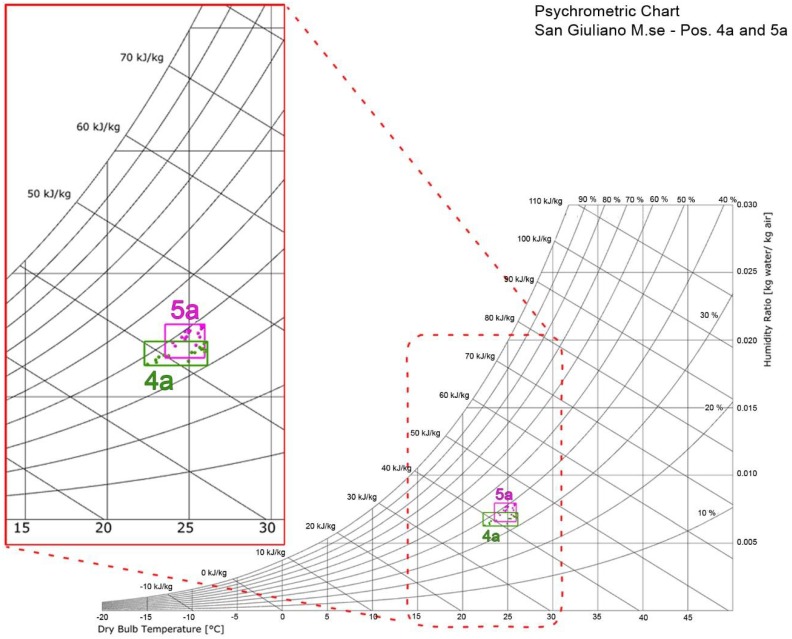
Example of personalized comfort zone based on the intersection of TSV = 0 data recorded for users 4a and 5a.

In this regard it is possible to consider, as hypothesis, two users, 5a and 4a, located in the same office (Figure 4). It is possible to identify an optimal TC level considering the intersection of the personalized comfort zones for the two users, by identifying the GCZa based on the user’s feedback.

As displayed above, the parametric model allows to calculate and to display the differences between the standard and personal comfort perception. The framework can use also the data collected by wearable and nearable devices to investigate the interactions between the variables or to provide predictions of the users, as described in the following paragraphs.

### 3.3. Dataset Definition and Machine Learning Approach

**Figure 5:**
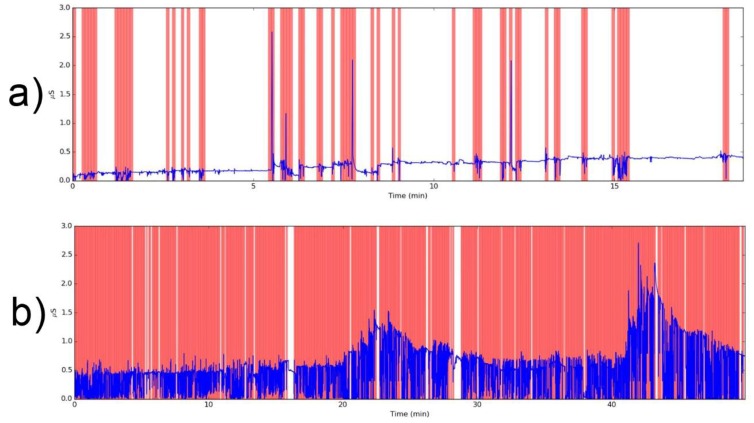
EDA comparison: (**a**) good series of data; (**b**) bad series of data.

All users have been informed about how to use the nearable and wearable devices and how to compile the web based survey. The recorded data by the wearable device have been verified and filtered considering a ML algorithm for automatically detecting EDA artefacts [51], generated from electronic noise or variation in the contact between the skin and the recording electrode caused by pressure, excessive movement, or adjustment of the device. The algorithm is available on a web page [52] or by downloading a Python script. The application of algorithm for the data check is essential to detect and filter noise and artefacts [52]. Through this algorithm the raw data, acquired with a sampling frequency of 4 Hz, are divided in periods of 5 s. Considering a binary classification, for all of these periods, a noise classification number equal to −1 (noise data, in red background in Figure 5), or 1 (clean data, in white background in Figure 5) is attributed.

Then a first dataset is defined, composed by the data of the nearable device aggregated with those of the wearable one elaborated with the EDA explorer algorithm. The result is a dataset structured considering 15,456 instances (rows) and 15 attributes (Time, *Z*-axis, *Y*-axis, *X*-axis, EDA_explorer_label, skin temperature-Tskin-, EDA, HR, Tair, RH, Trad, air velocity, CO_2_ concentration, illuminance level and User). Then a new dataset was built excluding: time dependencies, subjective variable related to the accelerations in the three axis, scarcely significant given the sedentary activities of the involved users, and environmental variable such as CO_2_ concentration and illuminance level. At present, the attention of the experimentation is paid on the environmental and subjective parameters that directly describe the TC conditions and allow the identification of user profile. Future developments will provide the analysis of the correlation between other environmental parameters, such as CO_2_ and illuminance level, and the thermal sensation through ML techniques.

Finally, the information related to T_air_ and T_rad_ are used for the calculation of T_o_ while the air velocity is excluded because the monitored values are closest to zero. The air velocity in the monitored spaces is closed to 0 m/s; for these reasons, this parameter is neglected. As a result, only 6 attributes (T_skin_ value, EDA, HR, To, RH and User) are considered and 9022 instances defined considering only the rows with a noise classification value equal to 1, as reported in the categorical column “EDA_explorer_label”. Table 8 summarizes the number of instances for each user and related data.

**Figure 6:**
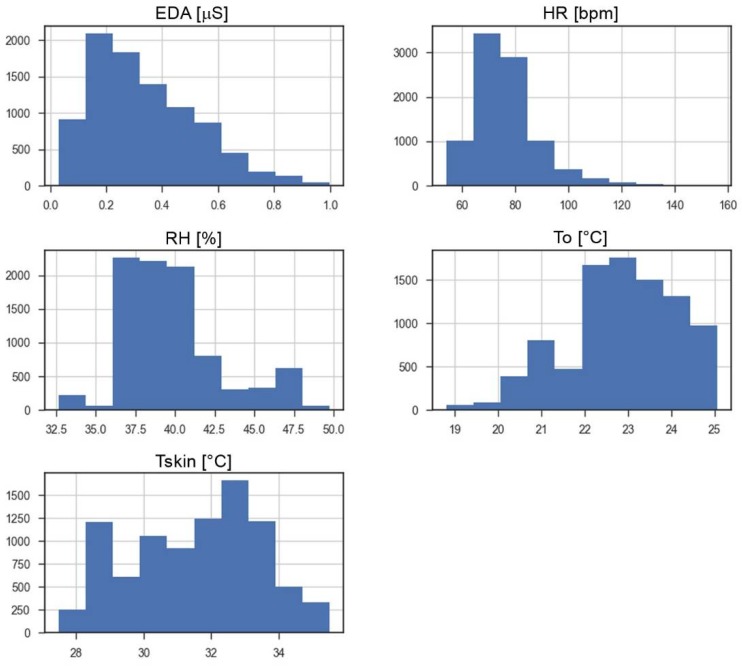
Histogram of each input variable.

Figure 6 reports the distribution of the input variables.

**Figure 7:**
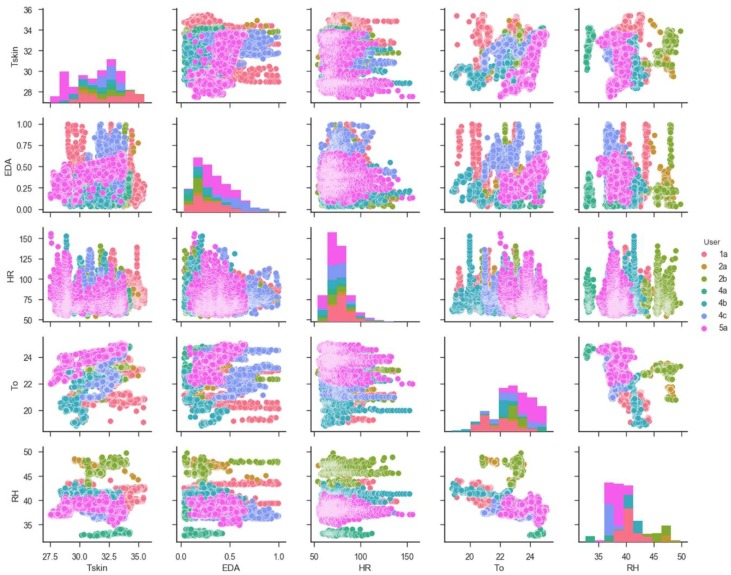
Interaction between the variables.

The data related to HR, EDA and skin surface temperature (T_skin_) have a pseudo-Gaussian distribution. Figure 7 reports the interaction between the variables. As reported in the legend, each color characterizes a specific user. Some pairs of attributes highlights a predictable relationship in some dimensions but, generally, it is not possible to identify which algorithms would be the best to validate and predict the users based on this dataset. For this purpose, a set of six different linear (Logistic Regression [53] and Linear Discriminant Analysis [54]) and non-linear (K-Nearest Neighbors [55], Classification and Regression Trees [56], Gaussian Naive Bayes [57], Support Vector Machines [58]) algorithms are considered. The dataset is divided into two subsets, composed by 80% and 20% of values. The former used to train the models and the latter for the validation. Table 9 reports the average accuracy (Avg.) and the standard deviation (St. dev.) of the simple linear and non-linear algorithms based on the training dataset. Depending on the scenario reported in Table 9, all instances of a different combination of the attributes from 0 to 4 (T_skin_, EDA, HR, T_o_ and RH) are considered as an input variable “x” and the instances of attribute 5 (User) as the target variable “y”. The metric of ‘accuracy’ is used to evaluate the models [59] defined as the ratio of the number of correctly predicted instances in divided by the total number of instances in the dataset. The k-fold cross validation (k = 10) [60] is used to evaluate the performances of the different algorithms on the dataset. Table 9 reports the Avg. and St. dev. for each algorithm evaluated 10 times (10 fold cross validation).

**Figure 8:**
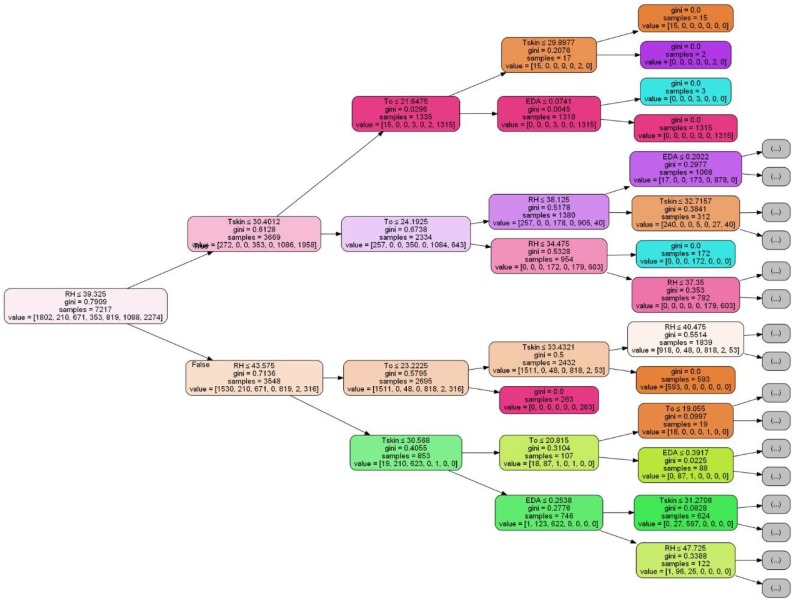
Visual representation of CART model for scenario V.

Among the models, the Classification and the Regression Trees (CART) algorithm has the highest estimation accuracy in all the considered scenarios (Table 9). CART is a non-parametric supervised learning method that predicts the value of a target variable by learning simple decision rules inferred from the data features [61]. Figure 8 reports the visual representation (truncated at fourth level for a better visualization) of the trained user classifier for the scenario V (see Table 9) that granted the highest average accuracy score, defined considering as input variables all the instances of T_skin_, EDA, T_o_ and RH columns.

In all level it is possible to note the “gini” impurity of the node, that reaches the zero value when all cases in the node fall into a single target category, the “sample” variable that indicates the number of samples at each node and, finally, the “value” as list of seven attributes, reports how many of the observation sorted into that node fall into each of seven categories (1a, 2a, 2b, 4a, 4b, 4c, 5a). In [62] the complete tree can be displayed thus allowing to visualize the rules extracted from the training dataset.

Identified the best model on the training dataset and visualized all the rules of classification, it is possible now to get an idea of the accuracy of the selected CART model and scenario V on the validation set, giving an independent final check on the accuracy of the selected model and the input variables in order to identify the users. Table 10 shows the classification report summarizing the results as a final accuracy score of the CART model directly on the validation set.

It shows the excellent results in term of prediction of each user considering four indicators [63]:

- Precision defined as a measure of a classifiers exactness;
- Recall considered as the completeness of the classifier;
- F1-score, a weighted average of precision and recall;
- Support, the number of occurrences of each label in y true.

The application of the ML approach has allowed to exclude the HR variable granting the highest level of accuracy for the specific case of sedentary activity. The ML approach allows then to identify the users and consequently their neutral TSV, considering objective and subjective variables.

## 4. Conclusions and Future Work

The standard approach for TC assessment is essentially based on a thermal physic model that does not consider any other factors (behavioral, physiological, and psychological) and the complex state of mind that could affect the TC perception. The developed framework combines the user’s feedback and an IoT-based solution with the functionality of parametric models and ML and allows one to overcome the limitations of the thermal energy balance equations. The comparison of all information acquired by survey highlights the differences between the individual perception of TC, TSV and GCZa, and those defined by the standards, PMV and GCZM, respectively. The wearable and nearable data elaborated with the functionality of ML, allow to investigate the possibility to find some dependences among the different variables in order to identify the different subjects. The proposed framework has allowed to detect the indoor environmental variables close to users, in addition to the biometric parameters and users’ feedback in order to:

- highlight differences among users and TC perception;
- define individual GCZa based on users’ feedback in order to optimize the TC control strategy;
- identify the most relevant parameters for users recognition and, consequently for their personal TC optimal perception identification;

The method is the basis for the development of individual control strategy that combines environmental variables and biometric with the powerfulness of ML techniques. The ML is applied to identify the users and their TC perception thus to overcome the limit of an imbalanced dataset due to a small number of users’ feedback in relation to the environmental and biometric data. A more balanced dataset, obtained through a longer detection of both environmental parameters and users’ feedback, will allow to directly predict the thermal sensation of the users. That is a goal of a future application of the framework. The adopted flexible solution can be used in different contexts (hospitals, schools, gyms, nursing homes, etc.) considering different type of users (divided by age, gender, presence of pathologies, etc.) as to identify possible useful pattern for the optimal management of personal thermal comfort. It can be upgraded with some other features including a more integrated approach that can consider also a chat bot following the user in the initial phase and in the activity of feedback recognition.

In the current case study, the influence of air velocity was neglected. The air velocity could become an important variable for thermal comfort assessment that cannot be excluded especially in indoor space with a forced air cooling/heating system [64,65]. The variability of the environmental parameters in the space deserves interest as they can be cause of thermal discomfort for the users. The current framework can face this issue by exploiting the flexibility and the reliability of the low cost devices integrated in a Wireless Sensor Network. Finally, the interaction between the environmental parameters, such as CO_2_ concentration and illuminance level, and the users’ thermal sensation will be investigated by exploiting the potential provided by ML techniques, thus allowing to include other aspects related to IEQ: Indoor Air Quality (IAQ), Indoor Lighting Quality (ILQ) [66,67,68], Acoustic comfort [69,70,71] and their interaction with the energy performance of buildings [72,73,74,75].

## Figures and Tables

**Table 1:** Characteristics of sensors used for TC assessment.

Sensor	Typical Range	Response Time	Accuracy
Relative humidity: capacitive humidity sensor	0 ÷ 100%	>2 s	±2%
Air temperature: thermistor	−40 ÷ +80 °C	>2 s	±0.5 °C
Radiant temperature: 10 k thermistor inside a 40 mm diameter hollow sphere, painted in matt black	−55 ÷ +60 °C	<10 s	±0.2 °C
Air velocity: low-cost hot wire anemometer	0 ÷ 27 m/s	<2 s	±4%

**Table 2:** Characteristics of sensors used for biometric data acquisition.

Sensor	Typical Range	Sampling Frequency
PPG sensor	-	64 Hz
EDA sensor	0.01 ÷ 100 µS	4 Hz
Skin Temperature sensor	−40 ÷ +85 °C	4 Hz
3-axes accelerometer	±2 g	32 Hz

**Table 3:** Dynamic insulation of clothing.

Workstation	Clothing Insulation [clo]
1a	0.98
2a	0.89
2b	1.01
3a	0.9
4a	0.94
4b	0.94
4c	0.91
5a	1.07

**Table 4:** Area of the office and personal data of the users involved in the test.

N.	Floor Area [m^2^]	User	Age [y]	Weight [kg]	Height [cm]	Gender [-]	Position [-]	Period of Test [-]
1	21.72	a	61	61.4	175	male	Senior researcher	II. 13–17 November 2017
2	41.94	a	39	81	178	male	Researcher	I. 6–10 November 2017
		b	35	85	179	male	Researcher	I. 6–10 November 2017
3	21.72	a	43	46	164	female	Researcher	III. 20–24 November 2017
4	20.69	a	29	60	160	female	Junior researcher	IV. 27–30 November 2017
		b	37	57	179	female	Researcher	III. 20–24 November 2017
		c	33	80.2	191	male	Technician	IV. 27–30 November 2017
5	20.26	a	35	70	177	male	Researcher	II. 13–17 November 2017

**Table 5:** Weather data for the test periods—minimum, average and maximum values of: external air temperature, relative humidity, solar radiation (diurnal average of solar radiation is from 9 a.m. to 5 p.m.), wind speed, rainfall (higher than 1.0 mm).

Period	External Environmental Variable	Min	Avg	Max	Days (Prec. > 1.0 mm)	Cumulative Precipitations [mm]
I. 6–10 November 2017	Air temperature [°C]	5.8	9.3	13.3	-	-
	Relative humidity [%]	76.3	98.1	99.7		
	Solar Radiation [W/m^2^]	4.5	102.8	409.2	-	-
	Wind speed [m/s]	0.2	1.4	2.6	-	-
	Rain [mm]	-	-	-	3/5	14.8
II. 13–17 November 2017	Air temperature [°C]	−0.1	6.2	14.3	-	-
	Relative humidity [%]	36.8	85.7	100.0		
	Solar Radiation [W/m^2^]	0.3	222.0	475.8	-	-
	Wind speed [m/s]	0.0	1.3	5.2	-	-
	Rain [mm]	-	-	-	0/5	0.0
III. 20–24 November 2017	Air temperature [°C]	0.6	7.2	14.3	-	-
	Relative humidity [%]	58.2	96.2	100.0		
	Solar Radiation [W/m^2^]	1.8	143.4	415.5	-	-
	Wind speed [m/s]	0.1	1.1	2.5	-	-
	Rain [mm]	-	-	-	0/5	0.0
IV. 27–30 November 2017	Air temperature [°C]	−3.2	2.5	11.4	-	-
	Relative humidity [%]	25.8	88.8	99.8		
	Solar Radiation [W/m^2^]	0.8	172.0	459.5	-	-
	Wind speed [m/s]	0.2	1.5	3.9	-	-
	Rain [mm]	-	-	-	1/4	1.8

**Table 6:** PMV_int_ and related range of PMV.

PMV_int_	PMV
3 (hot)	>2.5
2 (warm)	2.5:1.5
1 (slightly warm)	1.5:0.5
0 (neutral)	−0.5:0.5
−1 (slightly cool)	−1.5:−0.5
−2 (cool)	−2.5:−1.5
−3 (cold)	<−2.5

**Table 7:** PMV_int_ vs. TSV.

Workstation	PMV_int_ vs. TSV Difference
1a	16.67%
2a	72.73%
2b	61.54%
3a	25.00%
4a	45.83%
4b	29.17%
4c	44.44%
5a	10.53%

**Table 8:** User and related variables.

User	Instances	Variable	Min	Avg	Max
1a	2240	EDA [μS]	0.031	0.303	0.999
		HR [bpm]	74	80	139
		T_skin_ [°C]	28.96	32.58	35.51
		RH [%]	37.35	40.41	44.15
		T_o_ [°C]	19.1	21.88	23.97
2a	276	EDA [μS]	0.111	0.264	0.866
		HR [bpm]	55	79	114
		T_skin_ [°C]	29.53	30.93	34.92
		RH [%]	44.3	47.32	48.5
		T_o_ [°C]	21.04	22.58	23.54
2b	855	EDA [μS]	0.035	0.194	0.988
		HR [bpm]	54	76	141
		T_skin_ [°C]	30.44	32.08	33.99
		RH [%]	42.95	46.25	49.75
		T_o_ [°C]	20.78	23.03	23.53
3a	0	EDA [μS]	-	-	-
		HR [bpm]	-	-	-
		T_skin_ [°C]	-	-	-
		RH [%]	-	-	-
		T_o_ [°C]	-	-	-
4a	453	EDA [μS]	0.03	0.137	0.418
		HR [bpm]	59	72	112
		T_skin_ [°C]	30.12	32.39	34.25
		RH [%]	32.65	35.24	38.6
		T_o_ [°C]	21.81	23.55	24.83
4b	1012	EDA [μS]	0.032	0.25	0.645
		HR [bpm]	57	76	153
		T_skin_ [°C]	28.35	30.84	33.43
		RH [%]	39.4	40.84	43.6
		T_o_ [°C]	18.8	21.74	22.98
4c	1335	EDA [μS]	0.145	0.605	0.996
		HR [bpm]	56	77	136
		T_skin_ [°C]	30.2	32.46	33.82
		RH [%]	36.2	37.07	39.9
		T_o_ [°C]	20.85	22.86	24.65
5a	2851	EDA [μS]	0.07	0.356	0.658
		HR [bpm]	55	74	156
		T_skin_ [°C]	27.48	30.33	33.88
		RH [%]	34.9	38.2	41.4
		T_o_ [°C]	22	23.82	25.06

**Table 9:** Algorithms and related accuracy.

	Scenario I		Scenario II		Scenario III		Scenario IV		Scenario V	
	Input Variables:		Input Variables:		Input Variables:		Input Variables:		Input Variables:	
	Tskin, EDA, HR, T_o_ and RH		Tskin, EDA, HR and T_o_		Tskin, EDA, HR and RH		Tskin, EDA and HR		Tskin, EDA, T_o_ and RH	
Algorithms	Avg.	St. dev.	Avg.	St. dev.	Avg.	St. dev.	Avg.	St. dev.	Avg.	St. dev.
Logistic Regression	0.81409	0.01097	0.66468	0.020608	0.658721	0.013551	0.50145	0.01582	0.821118	0.015817
Linear Discriminant Analysis	0.834002	0.014409	0.679365	0.014593	0.712757	0.014929	0.508934	0.016283	0.837188	0.016283
K-Nearest Neighbors	0.939725	0.009485	0.807953	0.016847	0.874745	0.014654	0.628515	0.003083	0.991965	0.003083
Classification and Regression Trees	0.991964	0.003655	0.96564	0.006938	0.966609	0.006322	0.809057	0.002703	0.993211	0.00266
Gaussian Naive Bayes	0.829985	0.012559	0.707909	0.02119	0.789527	0.011923	0.537479	0.011854	0.809613	0.011854
Support Vector Machines	0.953167	0.009965	0.803516	0.025457	0.879319	0.019446	0.62186	0.005874	0.980602	0.005874

**Table 10:** Report.

User	Precision	Recall	F1-Score	Support
1a	1.00	0.99	0.99	438
2a	1.00	0.98	0.99	66
2b	0.99	1.00	1.00	184
3a	-	-	-	-
4a	1.00	1.00	1.00	100
4b	0.97	0.98	0.98	193
4c	1.00	1.00	1.00	247
5a	0.99	1.00	1.00	577
Avg/tot	0.99	0.99	0.99	1805

## Nomenclature

AM	Adaptive method
CART	Classification and Regression Trees
EDA	Electrodermal Activity
GCZa	adapted Graphic Comfort Zone
GCZM	Graphic Comfort Zone Method
HR	Heart Rate [bpm]
IAQ	Indoor Air Quality
IEQ	Indoor Environmental Quality
ILQ	Indoor Lighting Quality
ML	Machine Learning
PMV	Predicted Mean Vote [-]
PPD	Predicted Percentage of Dissatisfied [%]
RH	Relative humidity [%]
Tair	Air Temperature [°C]
TC	Thermal Comfort
To	Operative Temperature [°C]
Trad	Radiant Temperature [°C]
Tskin	Skin surface Temperature [°C]
TSV	Thermal Sensation Vote [-]
Vair	Air Velocity [m/s]
